# Unlocking Medical Breakthroughs: The Transformative Role of Case Reports in Clinical Discovery

**DOI:** 10.3390/reports8040216

**Published:** 2025-10-27

**Authors:** Toshio Hattori

**Affiliations:** Graduate School of Public Health, Shizuoka Graduate University of Public Health, Shizuoka 420-0881, Shizuoka, Japan; toshio.hattori.a3@tohoku.ac.jp

## 1. Introduction

Case reports are fundamental tools that allow clinicians to understand patients scientifically. They offer a unique opportunity to document rare or novel conditions and test hypotheses. Historically, case reports have led to the discovery of diseases such as adult T-cell leukemia (ATL) and acquired immune deficiency syndrome (AIDS), showcasing their transformative impact. In this editorial, I primarily describe how case reports or clinical studies have contributed to medical research based on my experiences. Case reports also have significant educational value, offering practical insights into decision-making, diagnostic challenges, and therapeutic outcomes for healthcare professionals; reports on rare diseases or novel treatments, in particular, foster innovation and problem-solving in clinical practice. For example, COVID-19 case reports highlighted the successful use of tocilizumab, demonstrating the complexity of inflammatory responses and emphasizing the need for personalized treatment approaches.

Moreover, case reports contribute to the discovery of new biomarkers for evaluating disease progression and treatment efficacy. Studies on galectin-9 (Gal-9) and osteopontin (OPN), for instance, have played a pivotal role in assessing the severity of infectious diseases, proposing these biomarkers as new indicators for disease management.

## 2. Human Retrovirus-Induced Diseases

### 2.1. Development of ATL Biology

**Figure 1:**
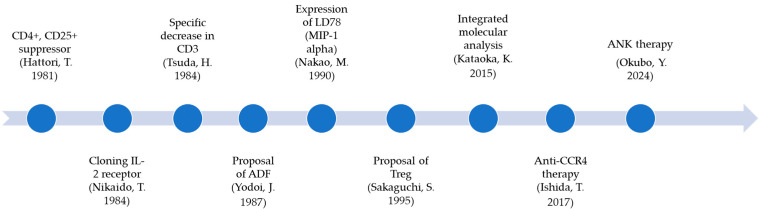
Parallel evolution of ATL cell biology and immunobiology.

The discovery of ATL depends on clinical specificity, but what supports it is the identification of the cells as T cells. Therefore, the efforts of immunologists to identify T cells have been widely endorsed [1]. Lymphocytic leukemia was reported to have a T cell phenotype in Japan [2]. Later, T-cell subsets were identified using particular monoclonal antibodies, leading to significant advancements in human T-cell research [3]. Researchers were able to distinguish the function from the specific antigens of T cell that had not been clearly defined until then. It was found that ATL cells, the first to be found to be involved in human retrovirus-induced leukemia, are CD4+ and CD25+ suppressor T cells [4]. This report highlights the period during which it became clear that the etiology of ATL is HTLV-1, attracting attention as the first retroviral cancer phenotype [5]. This discovery marked the beginning of immunobiology based on ATL biology (Figure 1). First, the IL-2 receptor gene was isolated from ATL cells, and the IL-2 receptor-inducing factor, ATL-derived factor (ADF), was characterized [6,7]. Furthermore, this discovery later significantly influenced the formation of the concept of Treg, leading to Tregs being differentiated by the expression of FoxP3 [8].

Furthermore, a novel chemokine was also found to be expressed in ATL cells [9]. This led to extensive studies of chemokines in ATL cells, and the specific expression of C-C chemokine receptor type 4 (CCR4) resulted in the development of a new therapy using anti-CCR4 [10].

The most mysterious aspect of ATL cell biology, which has been studied in various ways, is the decrease in T-cell receptor (TCR) expression, and it has been suggested that this stimulation of TCR contributes to leukemogenesis [11]. A recent extensive molecular analysis of ATL demonstrated frequent genetic alterations affecting components of TCR signaling and its downstream pathways, particularly the TCR–NF-κB signaling pathway [12,13]. However, it is unclear what is stimulating ATL’s TCR.

HTLV-1 encodes a Tax, a unique oncoprotein that activates various cellular and viral genes including cytokines and their receptors [14]. These cellular genes include costimulatory signal-related genes (CD80, CD86, and CD137L), which are known to stimulate natural killer (NK) cell activity. The expressed costimulatory antigens effectively stimulate cytotoxic T lymphocytes and natural killer cells [14]. Based on these findings, ANK therapy—in which natural killer (NK) cells are harvested from cancer-bearing patients and then activated, proliferatively amplified, and autologously administered—has been successfully conducted [15].

### 2.2. AIDS/TB Pathophysiology and Social Impact

The emergence of immunodeficient individuals with Pneumocystis pneumonia in Los Angeles marked the beginning of the AIDS era [16]. It has also been reported that some AIDS patients have antibodies against HTLV-1 [17]. However, it became clear that ATL patient serum does not recognize HIV, and although HIV and HTLV-1 are both retroviruses, they are distinct from each other [18].

The origin of HIV was proposed to be Africa, and the sub-Saharan African region carries the most significant burden of this pandemic. The risk of tuberculosis (TB) is increased during all stages of HIV infection, from about 10% over a lifetime in HIV-uninfected individuals to as high as 30% per annum in patients with advanced HIV infection [19,20]. The risk of TB increases with the duration of mining exposure. Zimbabwe is currently experiencing a high burden of TB, silicosis, and HIV among artisanal and small-scale miners [21]. Interferon-gamma release assays (IGRAs) have become a powerful tool for the diagnosis and evaluation of the therapeutic effect of TB [22,23]; however, since it is a measurement method using T-cell function, careful handling of the measurement results is recommended for application to HIV-infected individuals [24,25]. Matricellular proteins such as OPN and Gal-9 have also been extensively studied to evaluate the severity of MTB infection. OPN levels were significantly higher in TB patients and correlated with the levels of IL-12 and IL-18 TH1 helper type [26,27].

**Figure 2:**
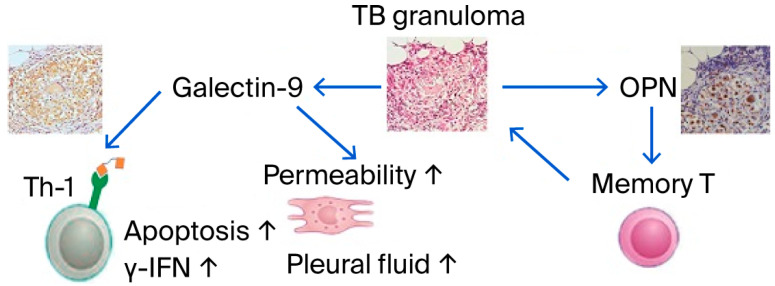
Roles of OPN and Gal-9 in TB pathology. Reprinted from Ref. [28].

The extensive study of a single patient with pleurisy revealed an abundant amount of Gal-9 in the pleural fluid, and Gal-9 induced γ-IFN production and apoptosis of lymphocytes (Figure 2) [28]. Gal-9 was also reported to activate latent HIV infection [29]. Moreover, OPN could be considered as a potential biomarker for tuberculosis surveillance and severity assessment [30]. Therefore, the roles of OPN and Gal-9 have been investigated in HIV/TB infection. Stronger positive and negative correlations of Gal-9 levels with viral loads and CD4 cell counts in HIV-infected patients, respectively, were observed than the OPN levels, indicating their association with HIV disease progression. Thus, significantly elevated Gal-9 levels were reported for the first time in HIV/TB co-infection and extrapulmonary tuberculosis in our study compared with single infections with HIV and tuberculosis [31]. 

The effects of SARS-CoV-2 infection on HIV/TB infection could be more complex. To understand the immunopathogenesis of this complex situation, the inflammatory score (INS) was assessed based on the percentage of reduction in the levels of inflammatory markers at the end of the treatment. However, the INS correlated negatively with SARS-CoV-2 seropositivity (*r* = −0.386, *p* = 0.039), indicating persistently raised inflammatory markers in these patients at the end of the treatment compared with seronegative individuals [32].

The results of clinical research have clarified that lifestyle diseases such as diabetes also affect diseases including AIDS and tuberculosis. Diabetic patients are also more likely to suffer from tuberculosis infections. An increase in HIV-infected individuals has also been observed, and it is necessary to closely monitor the impact of these factors on tuberculosis in the future, because medicines for HIV (62%) and tuberculosis (59%) are most frequently included in low- and middle-income countries and diabetes (86%) in high-income countries in the National Essential Medicines List [33].

## 3. Disaster/Tropical Infectious Diseases

In March 2011, the city of Sendai was hit by a devastating tsunami, which was caused by a magnitude 9.0 earthquake [34]. Since then, a disaster infectious disease control seminar has been held every year, and it has become clear that measures for tropical infectious diseases and disaster infectious diseases have many commonalities. Many of the discussed contagious diseases are caused by RNA viruses [35].

### 3.1. From Mosquito-Borne Diseases to COVID-19

Dengue and chikungunya are frequent causes of acute febrile illnesses in Southeast Asia and South America [36]. Many travelers to Thailand suffer from dengue after returning to their home country [37].

**Figure 3:**
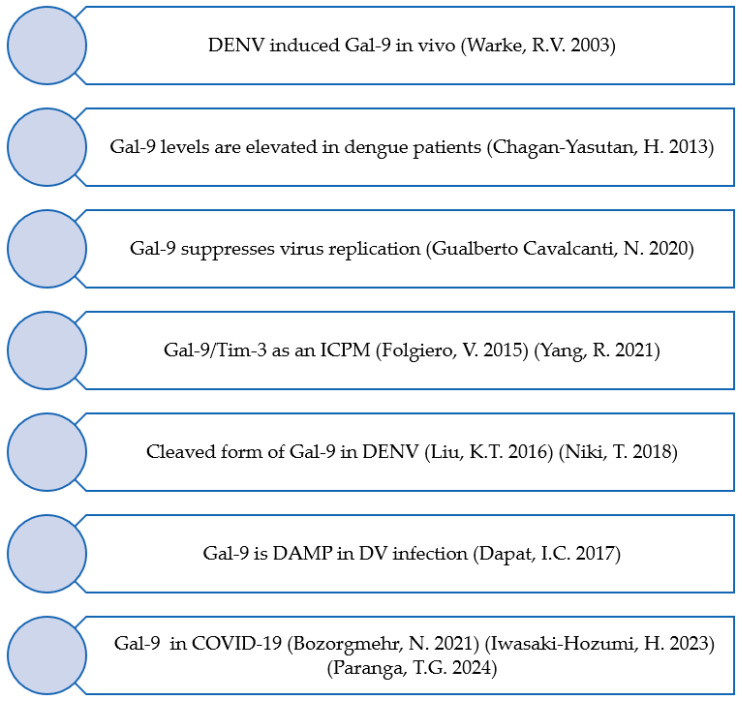
Development from Gal-9 dengue fever research to the immune checkpoint molecules.

Dengue virus (DENV) infection experiments have previously been performed using human umbilical endothelial cells as target cells. Differential display reverse transcription-PCR (DD-RTPCR) identified eight genes, including Gal-9, upon infection (Figure 3) [38]. We have described full-length Gal-9 (FL-Gal9) as a severity marker for dengue for the first time. The levels were higher in dengue hemorrhagic fever than in dengue fever and declined in the recovery phase. The levels were associated with DENV inflammatory responses and viral load [39]. Later, the increase in Gal-9 in the plasma of dengue patients was subsequently confirmed by other research groups using different testing kits [40]. In chikungunya patients, similar arbovirus-infected diseases and higher levels of Gal-9 compared with the controls’ median were found, but the levels of Gal-3 were lower than those of the controls. There was no statistical difference in the levels of Gal-1, -4, and -7 between patients and the control groups, indicating that Gal-9 plays a vital role in acute virus infection [41].

It was also revealed that some of the ELISA kits measure not only the full-length type but also the truncated type of Gal-9 [42]. Furthermore, DENV-infected THP-1 cells release Gal-9 as a danger-associated molecular pattern, and the released molecule may inhibit further virus replication [43]. DENV-infected dendritic cells produced Gal-9, which may facilitate their migration toward, for example, lymphoid organs. However, the pathological roles of dendritic cells heading toward the lymph nodes are not clear [44]. To clarify the biological roles of Gal-9, Gal-9 knockout mice were used. The mice mounted a more robust and vigorous virus-specific immune response in both acute and chronic viral infections, resulting in rapid viral clearance. In line with this observation, blocking Gal-9 signals to Tim-3-expressing T cells results in improved immune response [45]. In AML, the produced Gal-9 interacts with Tim-3 and induces indoleamine 2,3-dioxygenase 1 (IOD1) from AML blasts, which downregulates NK cell activity [46]. Gal-9 interacts with PD-1 and TIM-3 to regulate T-cell death and contributes to the persistence of exhausted T cells in the tumor microenvironment [47].

These findings have led to Gal-9 being identified as a member of the immune checkpoint molecules [46,48,49].

#### COVID-19

**Figure 4:**
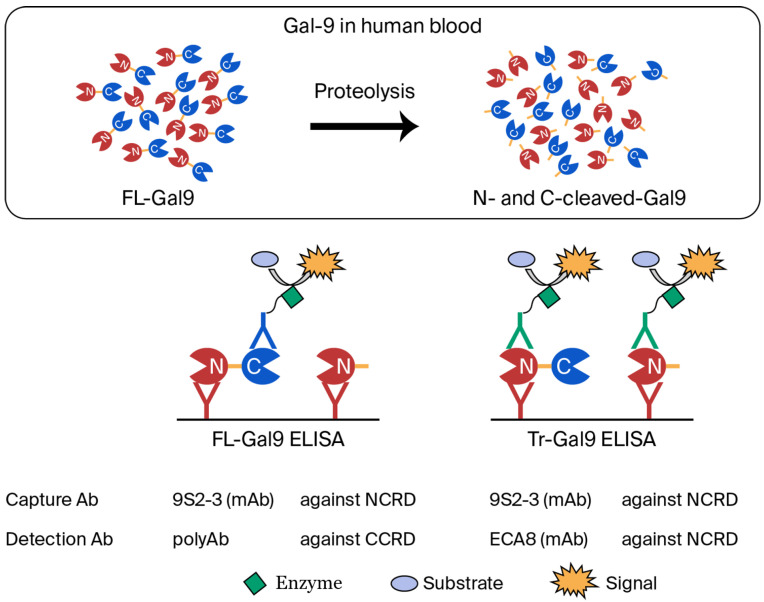
Two ELISA systems used to calculate N-cleaved Gal-9 (Tr-Gal9/FL-Gal9). Reprinted from Ref. [54].

The emergence of COVID-19 vividly illustrated the reality of disasters caused by acute viral infections. *Reports* organized a Special Issue on COVID-19 twice, published numerous papers, and released a book consisting of these papers [50]. The inflammation of COVID-19 is understood as a primary symptom, as explained by the concept of a cytokine storm. The administration of drugs as anticoagulants and anti-cytokines is recommended for COVID-19 treatment with these inferences. The anti-interleukin-6 receptor antibody tocilizumab (TCZ) appears to be an effective treatment option in COVID-19 patients with a risk of cytokine storms [51]. One case was reported in which a patient, exhibiting a strong inflammatory response, was administered TCZ. The following day, the fever subsided, and the inflammatory response gradually subsided. On the other hand, there has been a report of a case that appears to have worsened due to TCZ. It should be noted that the patients’ laboratory data (white cell count, ferritin, d-Dimer, C-reactive protein, and β_2_-microglobulin) did not increase, even at the time of exacerbation, except for Gal-9 [50]. Among 10 ICIs, Gal-9 levels play a prominent role in discriminating between survivors and non-survivors. Furthermore, both suPAR (soluble urokinase plasminogen activator receptor) and Gal-9 are elevated in severe COVID-19 cases and are associated with hyperinflammatory states. suPAR reflects immune activation and systemic inflammation, while Gal-9 contributes to immune suppression and cytokine storms [52]. It is noteworthy that neutrophils from COVID-19 patients, regardless of disease severity, secrete higher levels of Gal-9 compared with those from healthy controls. Therefore, it has been suggested that neutrophils are a potential source of soluble Gal-9 in the plasma of COVID-19 patients [53]. The Gal-9 levels studied here measured both FL-Gal9 and Tr-Gal9. The levels of N-cleaved Gal-9 were calculated by subtracting the concentration of FL-Gal9 (measured by FL-Gal9 ELISA) from the concentration of Tr-Gal9 (measured by Tr-Gal9 ELISA) (Figure 4). N-cleaved Gal-9 was found to be a prominent marker for the severity of COVID-19, and MMP-9 was involved in the cleavage [54].

People living with HIV (PLWH) with low CD4 counts are at higher risk of severe COVID-19 outcomes. Proteomics analysis showed that proteins such as complement factor D (CFD), vitronectin (VTN), and coagulation factors were significantly altered, indicating hypercoagulation and inflammation [55]. PLWH were hospitalized more frequently than the HIV-seronegative group and had a higher overall mortality rate, but once hospitalized, they had similar mortality rates. Older age, multimorbidity, and insurance status are associated with more severe outcomes among PLWH, suggesting the importance of targeted interventions to mitigate the effects of modifiable inequities [50]. ICI-treated cancer patients with COVID-19 were also studied because cancer patients are more susceptible to COVID-19 due to their compromised immune systems from both the cancer itself and its treatments. ICIs restore immune competency, which could influence COVID-19 outcomes. They found that the pooled event rate (PER) of COVID-19-related mortality was 39.73%, indicating a significant risk in this patient group. The PER of COVID-19-related pulmonary complications was 40.41%, and the need for ventilators was 34.92%, highlighting the severity of respiratory issues [50]. Patients in long-term, palliative, and hospice care are at increased risk of a severe course of COVID-19. Representatives of patients and their relatives must be involved in the development of appropriate support strategies to ensure that the measures implemented consider the needs of the patients and their relatives as effectively as possible [50].

Dementia patients are more likely to be discharged to hospice care or LTCFs and less likely to be discharged to recovery hospitals compared with those without dementia. It was concluded that dementia patients face systemic barriers to recovery-focused care, and healthcare systems should prioritize equitable discharge planning to improve the outcomes for this vulnerable population [56].

## 4. Conclusions

Case reports are the foundation of medical science, driving discovery and innovation. New fields may also be developed based on clinical findings. Therefore, case reports, as a tool allows one to create new insights, as demonstrated by the journal *Reports*, remain vital for documenting rare conditions, advancing therapeutic strategies, and enhancing global health knowledge. Clinicians and researchers should continue to embrace case reports as a powerful tool for scientific progress.
